# An Unusual Pulmonary Vascular Connection Encountered During Right Upper Lobectomy

**DOI:** 10.1093/icvts/ivag205

**Published:** 2026-07-22

**Authors:** Temmuz Baran Şencan, Mariam Arafa, Fikri Tarık Arıcı, Cansel Atinkaya Baytemir

**Affiliations:** Department of Thoracic Surgery, Medeniyet University Goztepe Training and Research Hospital, TC Goztepe Prof Dr Suleyman Yalcin City Hospital, 34722 Istanbul, Turkiye; Department of Thoracic Surgery, Medeniyet University Goztepe Training and Research Hospital, TC Goztepe Prof Dr Suleyman Yalcin City Hospital, 34722 Istanbul, Turkiye; Department of Thoracic Surgery, Medeniyet University Goztepe Training and Research Hospital, TC Goztepe Prof Dr Suleyman Yalcin City Hospital, 34722 Istanbul, Turkiye; Department of Thoracic Surgery, Medeniyet University Goztepe Training and Research Hospital, TC Goztepe Prof Dr Suleyman Yalcin City Hospital, 34722 Istanbul, Turkiye

**Keywords:** pulmonary vascular anomaly, arteriovenous connection, thoracic surgery, lung cancer

## Abstract

**Background:** Pulmonary vascular variations may complicate thoracic surgery, particularly video-assisted procedures, where such anomalies increase the risk of bleeding.

**Case presentation:** We report a 77-year-old female undergoing right upper lobectomy for invasive mucinous adenocarcinoma. Imaging revealed a 2 cm posterior right upper lobe nodule with low FDG uptake. Histology showed mixed adenocarcinoma and large-cell neuroendocrine carcinoma (stage IA). Intraoperatively, a vascular connection consistent with an arteriovenous fistula between the superior pulmonary vein and the interlobar pulmonary artery was identified and ligated.

**Conclusions:** Recognition of rare pulmonary vascular anomalies through careful preoperative imaging and intraoperative assessment is essential to minimize surgical complications and ensure safe thoracic resection..

## INTRODUCTION

Anatomical variations of pulmonary vessels may pose unexpected challenges during lung surgery. This is particularly relevant in procedures with limited visualization, such as video-assisted thoracoscopic surgery (VATS), where vascular anomalies may cause severe bleeding or necessitate conversion to thoracotomy.^1^ The reported incidence of pulmonary vascular anatomical variations is approximately 5%-15%.^2^ Therefore, careful preoperative imaging, particularly 3D CT angiography reconstructions, and heightened surgical awareness are of great importance.^3^ Rare and unusual vascular variations are typically reported as isolated case reports in the literature.^4^ Herein, we present an unusual vascular connection consistent with an arteriovenous fistula between a superior pulmonary vein branch extending toward the fissure and the interlobar pulmonary artery, which to our knowledge and following a review of the available English-language literature, has not been previously described.

## CASE PRESENTATION

A 77-year-old female presented with complaints of cough and back pain. Her medical history was significant for coronary artery disease, diabetes mellitus, and hypertension. Her surgical history included coronary artery bypass grafting and coronary stenting.

**Figure 1.**
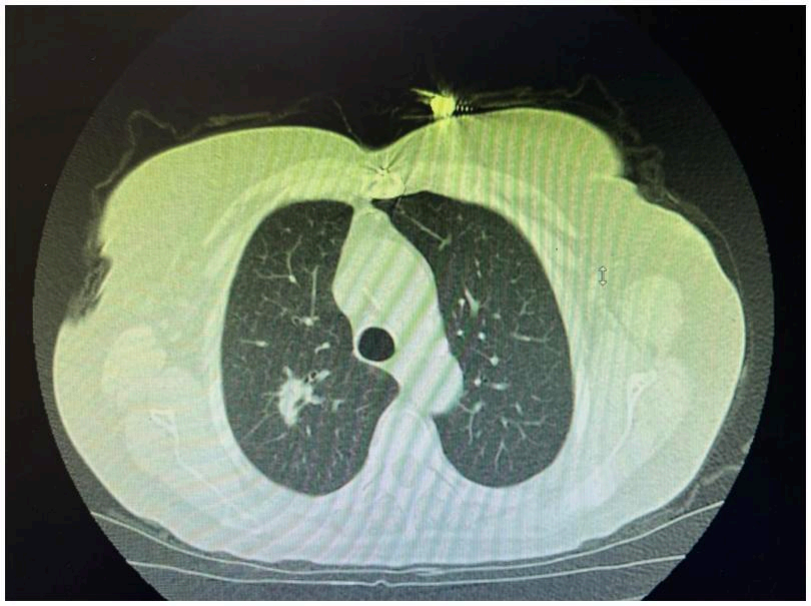
Axial chest CT image showing a nodular lesion in the posterior segment of the right upper lobe.

Chest CT revealed a lobulated nodular lesion, approximately 2 cm in diameter, located in the posterior segment of the right upper lobe (**Figure 1**).

PET-CT showed no FDG uptake in the lesion (SUVmax ∼2.3) and no pathological uptake in mediastinal lymph nodes or distant organs. Transthoracic needle biopsy confirmed invasive mucinous adenocarcinoma. In December 2024, a right upper lobectomy via thoracotomy with systematic mediastinal lymph node dissection was performed.

**Figure 2.**
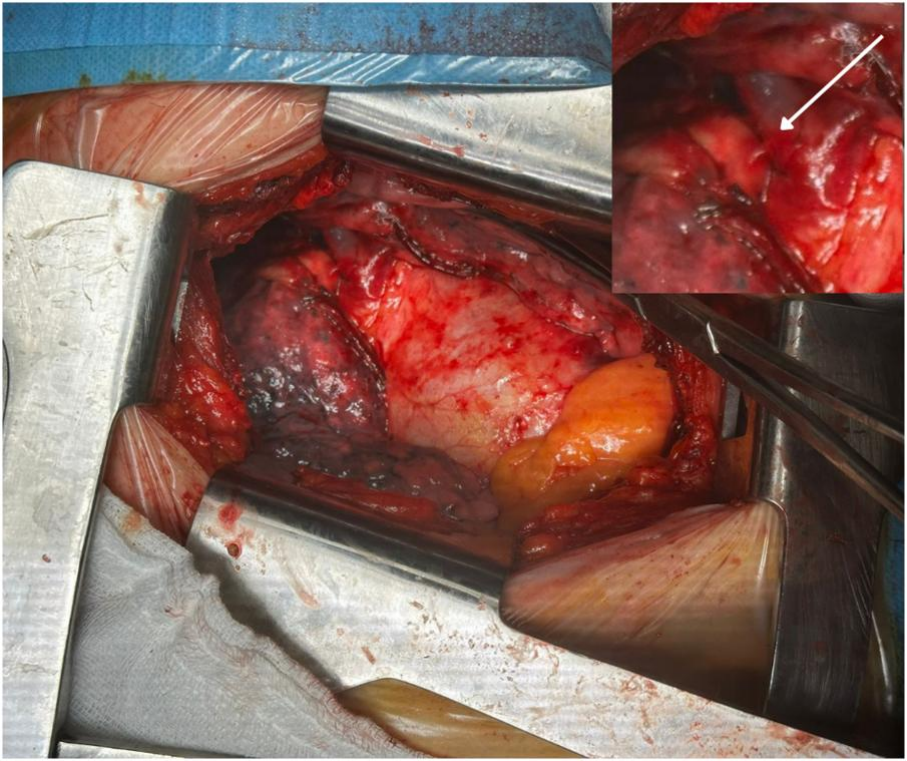
Intraoperative photograph showing an abnormal vascular connection consistent with an arteriovenous fistula between the superior pulmonary vein and the interlobar pulmonary artery before ligation.

Intraoperatively, a vascular connection consistent with an arteriovenous fistula was observed between the right superior pulmonary vein and the interlobar pulmonary artery (**Figure 2**).

The vascular connection was carefully dissected, controlled, and safely ligated without intraoperative complications. Pathological analysis revealed a mixed-type tumor (2.1 cm) composed of 75% non-mucinous adenocarcinoma (poorly differentiated), 15% mucinous adenocarcinoma (moderately differentiated), 10% large-cell neuroendocrine carcinoma (high grade). The pathological stage was pT1c N0 M0 (stage IA).

The patient developed intraoperative bradycardia, which was treated with atropine, followed by transient inotropic support. She was transferred to the ICU. Postoperative atelectasis was managed with bronchoscopy and BPAP. On postoperative day 4, she developed NSTEMI and was managed in the cardiology ICU. She was later transferred to the ward and remained stable at 8-month follow-up.

## DISCUSSION

Pulmonary vascular anomalies may be congenital or acquired and can involve anomalous arterial branching, abnormal venous drainage, or arteriovenous communications. Although many remain asymptomatic, recognition of these variations is important in thoracic surgery because unrecognized vascular anomalies may increase the risk of intraoperative complications. The anomaly coursed between the superior pulmonary vein and the interlobar pulmonary artery. Failure to recognize this anomaly intraoperatively could have resulted in severe bleeding. Indeed, life-threatening complications have been reported when anomalous pulmonary venous confluences were inadvertently ligated; for example, accidental resection of a common trunk of the left upper and lower pulmonary veins during lobectomy.^1^

A similar variation, in which the superior segmental vein of the right lower lobe drained anomalously into the right superior pulmonary vein, was described by Amore et al^4^ Pawlica et al^1^ also reported cases of anomalous segmental arterial origins in the right lung. Preoperative imaging in our case was limited to contrast-enhanced thoracic CT, and no vascular anomaly was detected prior to surgery. Following the intraoperative finding, the CT images were retrospectively reevaluated by 2 independent radiologists; however, the vascular connection could not be clearly demonstrated. This may be explained by the limitations of CT angiography, as contrast material is administered via the venous route and arterial pressure exceeds venous pressure, potentially limiting contrast passage from arterial to venous structures. Although the anomaly was not identifiable in our patient, advanced imaging techniques such as 3D CT angiographic reconstruction may improve the detection of unusual pulmonary vascular anatomy. In addition, dedicated preoperative review of vascular structures in collaboration with experienced thoracic radiologists may help reduce the risk of unexpected intraoperative findings and vascular injury.

Mucinous adenocarcinomas often demonstrate low FDG uptake on PET due to their high mucin content, which may result in false-negative findings.^5^ In our case, the lesion also showed low SUV values, consistent with this characteristic. Despite the presence of a large-cell neuroendocrine carcinoma component, the PET uptake remained low, likely due to the small size of this component.

Pulmonary arteriovenous fistulas are clinically significant vascular anomalies that may cause right-to-left shunting, hypoxemia, and embolic events. In the present case, the observed vascular structure was considered most consistent with a presumed arteriovenous connection.

This case has several limitations. Angiographic and histopathological confirmation were not available, and the vascular structure could not be definitively characterized. Therefore, the finding should be interpreted as a vascular connection consistent with an arteriovenous fistula rather than a confirmed diagnosis.

## CONCLUSION

Thorough evaluation of preoperative imaging and anticipation of possible vascular variations are critical for safe lung surgery. Rare anatomical anomalies, if unrecognized, may result in serious intraoperative complications. In our patient, the vascular anomaly was extremely subtle and could not be detected on routine preoperative imaging; therefore, careful intraoperative dissection was essential to prevent major complications.

## Data Availability

The data underlying this article are available in the article and in its online supplementary material.

## References

[1] Pawlica MT , Buchajska K, Gabrysz Z, et al Clinically important pulmonary vascular variations: a narrative review. J Thorac Dis. 2024;16:3406-3421. 10.21037/jtd-23-1715 38883672 PMC11170393

[2] Yamada S , Suga A, Inoue Y, Iwazaki M. Importance of preoperative assessment of pulmonary venous anomaly for safe video-assisted lobectomy. Interact CardioVasc Thorac Surg. 2010;10:851-854. 10.1510/icvts.2009.221804 20332219

[3] Matsuda M , Mizuuchi H, Ito K, Kouso H. En bloc right upper bilobectomy for a patient with incomplete minor fissure and multiple anomalies of the right superior pulmonary vein. Cureus. 2024;16:e70080. 10.7759/cureus.70080 39463670 PMC11503712

[4] Amore D , Muto E, Caterino U, et al Anomalous segmental pulmonary vein: additional V6 behind the bronchus intermedius draining into the superior pulmonary vein. Monaldi Arch Chest Dis. 2022;92:2196. 10.4081/monaldi.2022.2196 35352543

[5] Shim SS , Han J. FDG-PET/CT imaging in assessing mucin-producing non-small cell lung cancer with pathologic correlation. Ann Nucl Med. 2010;24:357-362. 10.1007/s12149-010-0358-x 20306161

